# Role of Rad18 in B cell activation and lymphomagenesis

**DOI:** 10.1038/s41598-024-57018-w

**Published:** 2024-03-25

**Authors:** Kevin Kalweit, Vanessa Gölling, Christian Kosan, Berit Jungnickel

**Affiliations:** Department of Cell Biology, Institute of Biochemistry and Biophysics, Faculty of Biological Sciences, Friedrich Schiller University Jena, Hans Knöll Strasse 2, 07745 Jena, Germany

**Keywords:** Biochemistry, Cancer, Cell biology, Genetics, Immunology, Molecular biology

## Abstract

Maintenance of genome integrity is instrumental in preventing cancer. In addition to DNA repair pathways that prevent damage to DNA, damage tolerance pathways allow for the survival of cells that encounter DNA damage during replication. The Rad6/18 pathway is instrumental in this process, mediating damage bypass by ubiquitination of proliferating cell nuclear antigen. Previous studies have shown different roles of Rad18 in vivo and in tumorigenesis. Here, we show that B cells induce Rad18 expression upon proliferation induction. We have therefore analysed the role of Rad18 in B cell activation as well as in B cell lymphomagenesis mediated by an Eµ–Myc transgene. We find no activation defects or survival differences between Rad18 WT mice and two different models of Rad18 deficient tumour mice. Also, tumour subtypes do not differ between the mouse models. Accordingly, functions of Rad18 in B cell activation and tumorigenesis may be compensated for by other pathways in B cells.

## Introduction

DNA damage repair is essential for cell survival and prevention of tumorigenesis^1^. Small or bulky DNA lesions are repaired by base and nucleotide excision repair. Errors during replication or DNA lesions are processed by mismatch repair. Double-stranded DNA breaks can be repaired by either non-homologous end joining or homologous recombination. Nevertheless, DNA lesions may persist into replication and would then stall the replication fork.

In this instance, a damage tolerance pathway, the Rad6 pathway, is activated^2^. The Rad6 pathway leads to ubiquitination of proliferating cell nuclear antigen (PCNA) at its K164 residue^3^. Monoubiquitination of PCNA, mediated by Rad6 together with the E3 ligase Rad18, leads to the recruitment of translesion polymerases, which can synthesize past bulky lesions since they have a flexible catalytic site that is able to tolerate such damage. The process is, however, potentially error-prone. Alternatively, error-free bypass of the lesion by a template switch of replication to the undamaged sister chromatid may be triggered by K63-linked polyubiquitination of PCNA at K164, mediated by Ubc13 together with MMS2 and Rad5 in yeast, or helicase-like transcription factor (HLTF) and SNF histone linker PHD RING Helicase (SHPRH) in mammals^3–6^.

The Rad6 pathway enables survival of DNA damage in proliferating cells. This is due to its function in PCNA ubiquitination, as well as independent functions in double strand break repair by homologous recombination^7^ and activation of the Fanconi anaemia pathway^8^. In vivo*,* Rad18 functions are particularly important during spermatogenesis, where Rad18 acts during meiosis^9^ and to maintain germline stem cell integrity during aging^10,11^. Also, Rad18 has been shown to play a role during viral infection^12,13^. Moreover, PCNA ubiquitination is important during somatic hypermutation of immunoglobulin genes in B cells^14–17^ and plays a role in haematopoiesis, independently of a previously discovered function in activation of the Fanconi anaemia pathway^18^.

Several studies have addressed the role of Rad18 in tumorigenesis^19^. Rad18 is responsible together with Polymerase κ for cells to overcome oncogene-induced replication stress^20^. Rad18 mediates specific mutational signatures and shapes the genomic landscape of carcinogen-induced tumours^21^. Rad18 plays an important role during establishment of chemoresistance of colorectal cancer cells^22^. In addition, Rad18 cooperates with MAGE-A4 overexpressed in cancer cells to mediate chemoresistance^23^. Rad18 also affects migration and invasion of oesophageal squamous cell cancer through the JNK-MMP pathway^24^ and promotes colorectal cancer metastasis by activating the epithelial-mesenchymal transition pathway^22^. A knockdown of Rad18 inhibited glioblastoma development^25^ and led to decreased resistance to ionizing radiation in glioma cells^26^. Accordingly, multiple roles for Rad18 function in other tumours may be envisioned.

A recent study analysed the role of Rad18 in induction of haematological malignancies by DMBA treatment^18^. The authors observed an increase in the generation of haematological malignancies in Rad18-deficient mice. Of particular importance is the tenfold increase of generation of B cell malignancies in the Rad18 deficient mice, implying that Rad18 might play a particular role in B cell malignancies.

We have therefore used the Eµ–Myc model to analyse the role of Rad18 in B cell lymphomagenesis. We observe no differences in tumour formation between WT and Rad18-deficient mice.

## Results

### Establishment of Rad18 mouse models

**Figure 1:**
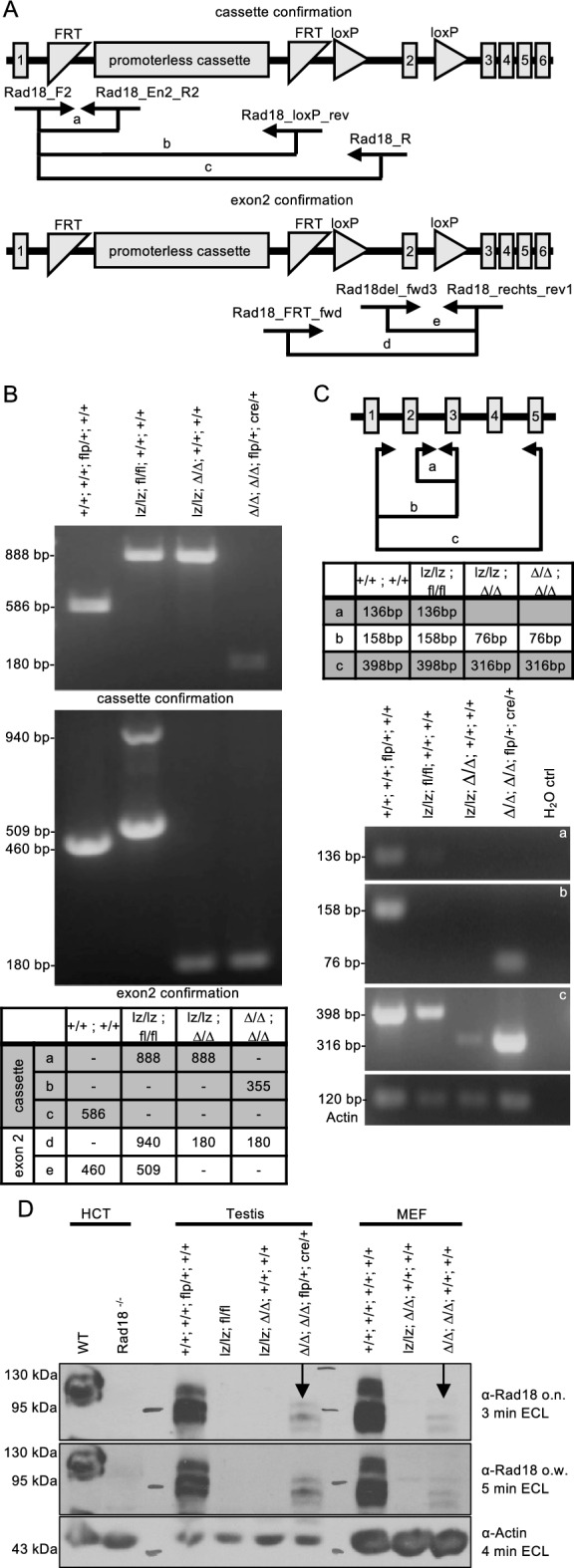
Molecular analysis of the RD and RDF mouse lines. (**A**) Schematic of the genetic background for Rad18 knockout with corresponding primers for genotyping PCR shown in (B). Squares with numbers indicate exons. (**B**) Representative genotype PCR. (**C**) Expression of Rad18 mRNA in mouse testis via RT-PCR with different primers. Actin was used as a loading control. (**D**) Western blot of testis tissue and MEF of both cell lines and the HCT116 cell line incubated with primary AB overnight (o.n.) or over weekend (o.w.). Actin was used as a loading control.

To analyse the role of Rad18, we used Rad18 mice available from the EMMA consortium. These mice harbour a lacZ expression cassette flanked by frt sites upstream of exon 2 of the Rad18 gene and have a loxP flanked exon 2 (Fig. 1A). Crossing of the mice with a Deleter Cre line (CMV-Cre) generated mice with the frt-flanked expression cassette and no exon 2 (Fig. 1A, henceforth called Rad18 DeleterCre: RD mice). Sequential crossing of the mice with first a mouse strain expressing the flp recombinase in all cells, and then the Deleter Cre strain generated mice that contain no expression cassette and no exon 2 (Fig. 1A, henceforth called Rad18 Deleter Cre Flp recombinase: RDF mice).

The genetic setup of the different mice after crossing, as assessed by PCR, was as expected (Fig. 1B, Suppl. Figure S2A), as was mRNA expression of Rad18 (Fig. 1C, Suppl. Figure S2B). Western Blot analysis of testis tissue showed that RD mice completely lost expression of Rad18, whereas the RDF mice showed a minor band at a somewhat reduced size, thereby indicating potential leakiness of the knockout by what may be downstream translation initiation on the Rad18 mRNA (Fig. 1D, Suppl. Figure S3). We therefore decided to perform all further analyses with both types of knockout mice, as in one case the presence of the selection cassette may be considered problematic, while in the other case the residual Rad18-like protein may mask an underlying phenotype.

### Rad18 expression in activated B cells

**Figure 2:**
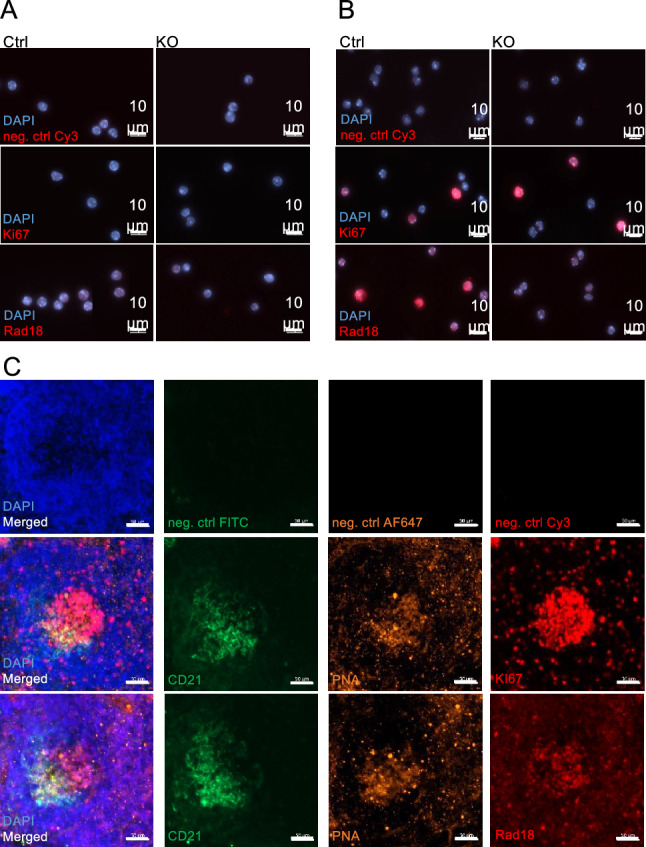
Expression of Rad18 in proliferating B cells. (**A, B**) Immunohistochemical staining of splenic B cells from *Rad18* WT and KO mice. After isolation and ensured purity, B cells were seeded onto Poly-L-lysine coated coverslips and PFA-fixed before IHC; depicted is a coloured overlay of representative microscope pictures of fixed WT and *Rad18* KO mouse B cells, either unstimulated (day 0, **A**) or stimulated for 3 days with anti-CD40/IL4 (**B**). Negative controls (neg. ctrl) were prepared by incubating only with the secondary antibody. Representative pictures of 3 independent experiments are shown. (**C**) Coloured overlay of representative single channel images of 10 µm-thick spleen sections from immunised WT mice. Negative controls were prepared by incubating only with the secondary antibody, n = 6 in total. Scale bar: 50 µm.

Rad18 is particularly important in proliferating cells. To analyse whether Rad18 is expressed in resting versus proliferating murine B cells, we isolated B cells from spleens by CD43 MACS depletion and cultured them in the presence of anti-CD40/IL4 for 3 days. As expected, proliferating cells were characterized by the expression of the proliferation marker Ki67 (Fig. 2A). Interestingly, only proliferating B cells expressed Rad18, while resting B cells stained on day 0 did not (Fig. 2A, B). In addition, we found Rad18 expression in germinal centres of mouse spleens, but not in the surrounding resting mantle cells (Fig. 2C). Western Blot analyses confirmed that Rad18 is expressed in activated but not resting B cells (Suppl. Figure S4). Accordingly, Rad18 is specifically expressed in proliferating B cells, and may thus play a role in B cell activation or lymphomagenesis.

### Role of Rad18 in B cell activation and class switch recombination

**Figure 3:**
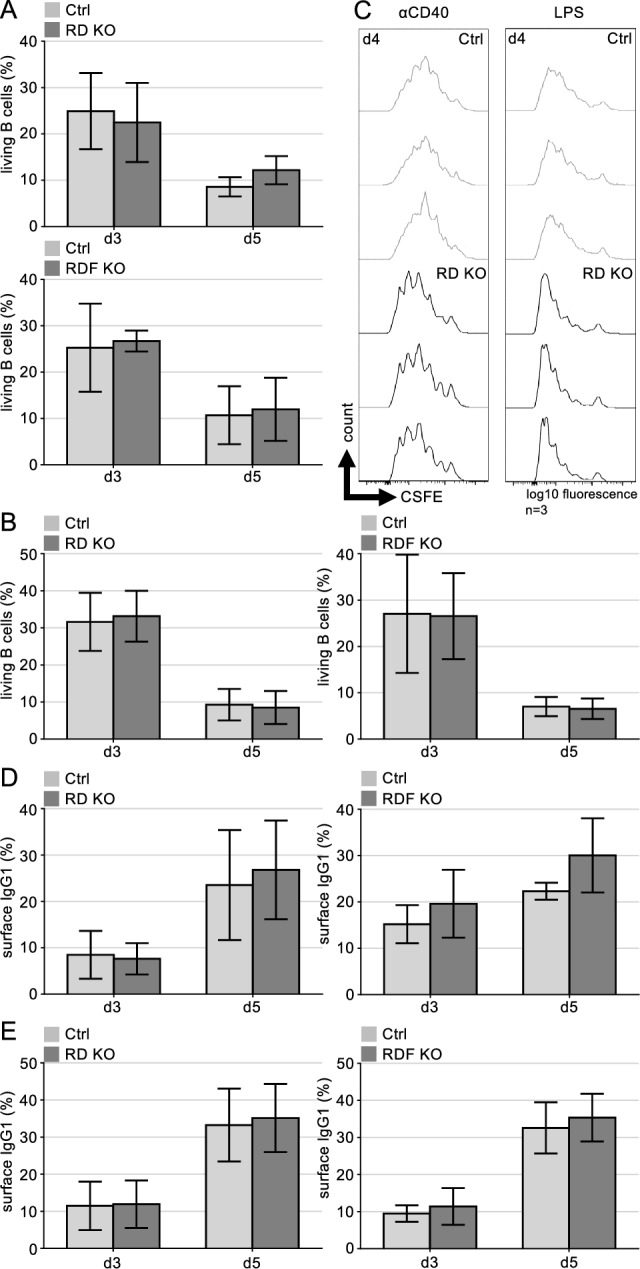
Normal activation and class switch recombination in Rad18 deficient B cells. (**A, B**) Living B cells out of total in percentage of (A) RD mice with n = 5 mice per genotype and RDF mice with n = 3 mice per genotype, stimulated with anti-CD40/IL-4 or (**B**) with LPS/IL-4. Statistics were calculated using a Students *t* test. *p* > 0.05. (**C**) FACS analysis of purified CSFE-stained B cells, stimulated either with anti-CD40/IL-4 or LPS/IL-4. Representative experiment of n = 3 mice per genotype, analysed in triplicates. **(D, E)** FACS analysis of surface IgG1 positive B cells in percentage of (**D**) RD mice with n = 5 mice per genotype and of RDF mice with n = 3 mice per genotype, stimulated with anti-CD40/IL4 or (**E**) with LPS/IL4. Statistics were calculated using a Students *t* test. *p* > 0.05.

To assess the role of Rad18 in B cell activation, we isolated B cells from spleens of WT and Rad18KO mice in the RD and RDF lines and stimulated them with anti-CD40/IL4 or LPS/IL4. Survival of B cells after stimulation was not different between the respective WT and Rad18KO mice (Fig. 3A, B), nor was the proliferation of the cells as assessed by CFSE staining (Fig. 3C). Also, class switch recombination to IgG1 was equally efficient for WT and Rad18 KO mice in both the RD and RDF line (Fig. 3D, E). Accordingly, Rad18KO mice did not show any evident phenotype in B cell activation.

### Role of Rad18 in B cell lymphomagenesis

**Figure 4:**
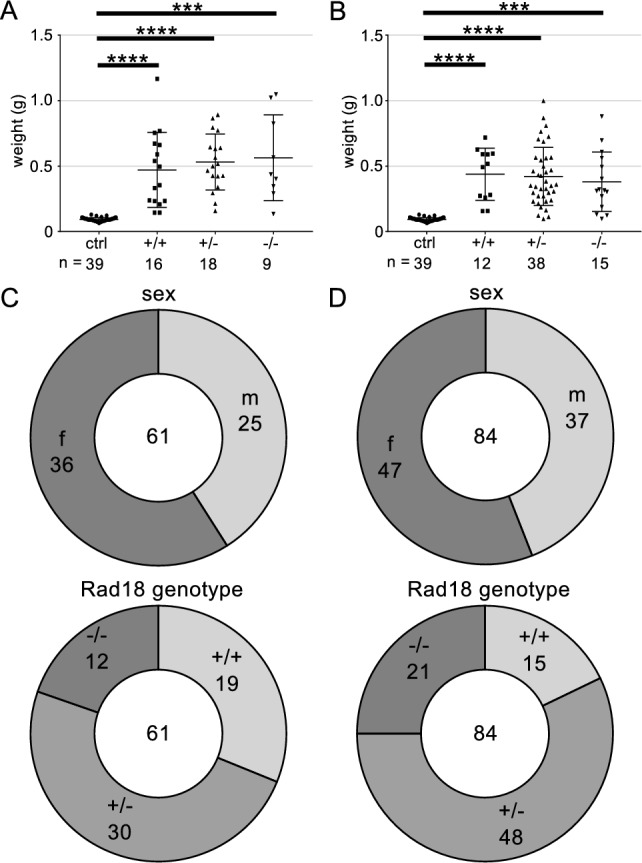
Splenomegaly caused by B cell tumours is independent of sex and Rad18 genotype. (**A**, **B**) Weight of spleens of *myc* transgenic mice of the RDM (**A**) and RDFM (**B**) mouse line after developing a tumour. Genotype indicate Rad18^+/+^, Rad18^+/-^ and Rad18^−/−^ mice; ctrl as a control are Rad18^+/+^ mice without transgenic *myc*. Each data point represents one analysed mouse per genotype. Numbers indicate total mice analysed per genotype. Statistics were calculated using Students *t* test comparing each possible genotypes individually. ***p ≤ 0.001, ****p ≤ 0.0001. (**C, D**) Absolute numbers of analysed mice of RDM (**C**) and RDFM (**D**) regarding sex and Rad18 genotype. The total number of analysed mice is given in the middle of the pie charts; fractions indicate sex or Rad18 genotype with corresponding absolute numbers of analysed mice.

To analyse the impact of Rad18 on tumour formation, we crossed RD and RDF mice with Eµ–Myc mice^27,28^, henceforth called RDM and RDFM mice. Both RDM and RDFM mice developed tumours starting at an age of 7 weeks and all tumour-bearing mice showed splenomegaly compared to control mice (Fig. 4A, B). Neither sex nor the two different Rad18-KO Eµ–Myc mouse models exhibited any difference in tumour development as assessed by spleen weight (Fig. 4C, D).

**Figure 5:**
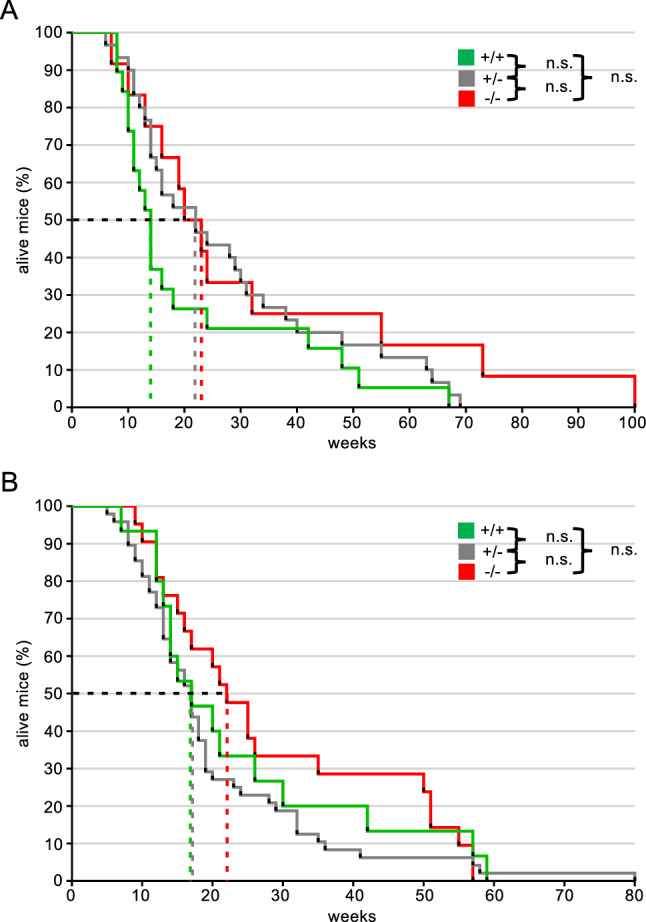
Survival and tumorigenesis in Rad18 deficient mice. (**A, B**) Survival curves of analysed RDM (**A**) and RDFM (**B**) mice, which developed B cell tumours. The threshold of 50% mortality rate is indicated with the black dotted line and with coloured dotted lines for each Rad18 genotype. For (**A**); n = 19 Rad18^+/+^, n = 30 for Rad18^+/−^ and n = 12 for Rad18^−/−^. For (**B**); n = 15 Rad18^+/+^, n = 48 for Rad18^+/−^ and n = 21 for Rad18^−/−^. Statistics were calculated with a Log-rank (Mantel-Cox) test and a Gethan-Breslow-Wilcoxon test using the GraphPad software. *p* > 0.05.

We therefore monitored survival of the RDM and RDFM mice to quantitatively determine the impact of Rad18 on tumour formation. Mice were checked regularly, and whenever evidence of tumour formation was detected by altered behaviour, accelerated breathing or lymph node swelling, they were sacrificed and analysed. Kaplan–Meier-plots of the total populations are shown in Fig. 5A, B. There were no statistically significant differences between the respective WT and Rad18-KO genotypes of both the RDM and RDFM line. Apparently, Rad18 does not affect tumour formation or progression in the Eµ–Myc model.

**Figure 6:**
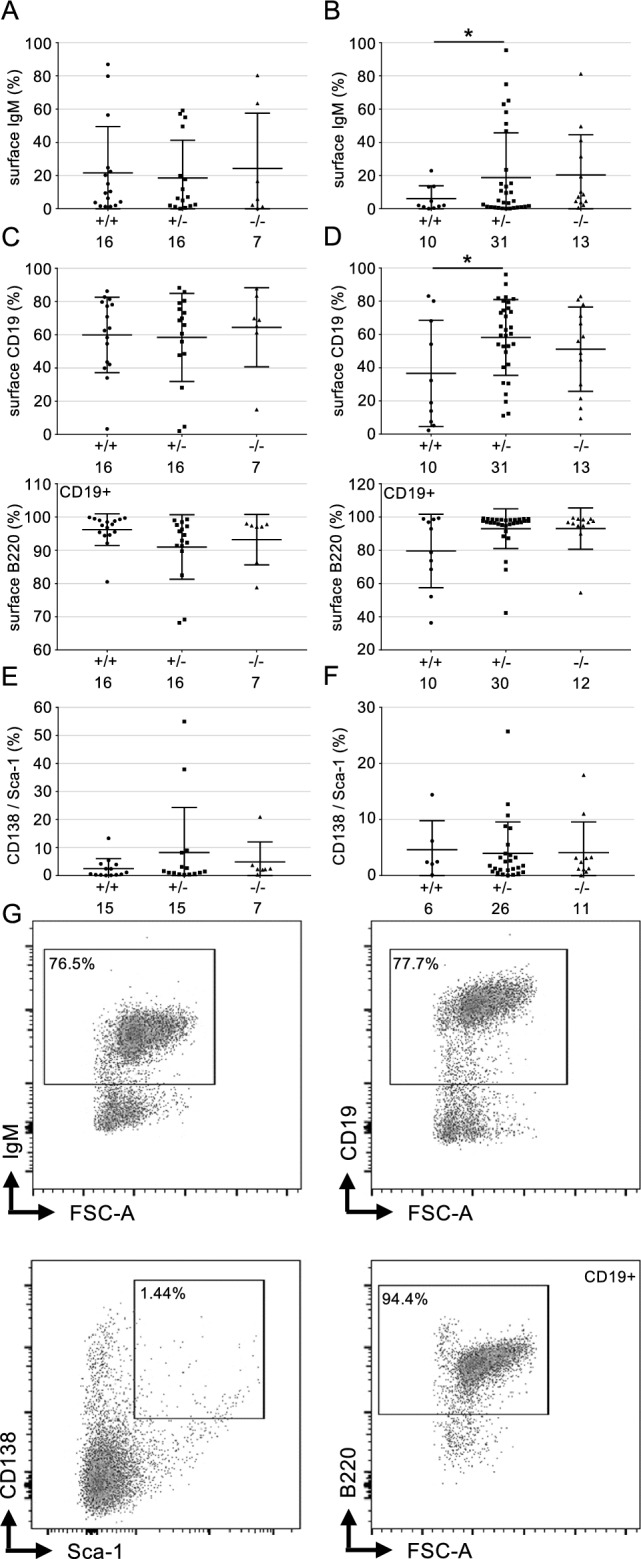
FACS analysis of splenic B cell tumours. (**A, B**) Surface IgM status of splenic B cells of the RDM (**A**) and RDFM (**B**) mice that have developed a B cell tumour. Each data point represents one mouse per genotype with absolute numbers given at the X-axis. Statistics were performed using a Students *t* test. *p ≤ 0.05. (**C, D**) Surface B220 and CD19 status of splenic B cells of the RDM (**C**) and RDFM (**D**) mice that developed a B cell tumour. B220 was pre-gated out of CD19 positive cells. Each data point represents one mouse per genotype with absolute numbers given at the X-axis. Statistics were performed using a Students *t* test. *p ≤ 0.05. (**E**, **F**) Plasma cell status of splenic B cells of RDM (**E**) and RDFM (**F**) mouse line developed a B cell tumour. Double positive cells for CD138 and Sca-1 are shown in the graphs to determine plasma cells. Each data point represents one mouse per genotype with absolute numbers given at the X-axis. Statistics were performed using a Students *t* test. *p* > 0.05. (**G**) Representative FACS analysis corresponding to (**A**–**F**) of a RDFM mouse spleen.

Finally, we analysed whether a Rad18-KO would affect the tumour phenotype. Both RDM and RDFM mice formed IgM+ and IgM− tumours to the same extent, thereby indicating mature and pre- or pro B cell tumours, respectively. However, there was no statistically significant difference between WT and Rad18-KO mice (Fig. 6A, B). Furthermore, we found no differences for CD19 and B220 expression in malignant B cells (Fig. 6C–G). Likewise, the extent of plasma cell tumour generation did not depend on Rad18 genotype (Fig. 6E, F). Taken together, we found no evidence that Rad18 has any impact on the phenotype of the B cell tumours formed, the speed of tumour formation or tumour progression.

## Discussion

In the present study, we have analysed the role of Rad18 in B cell activation and lymphomagenesis. Although we observed specific expression of Rad18 in proliferating B cells, we found no difference in the activation of B cells or the survival of Eµ–Myc -induced tumours in WT versus Rad18KO mice.

We have shown that Rad18 is specifically expressed in proliferating but not resting B cells. Rad18 has been shown to be an E2F3 target^29^, and is thus induced in cells during proliferation. Accordingly, in most somatic cells, Rad18 is not expressed, and it would only be expressed and active in stem cells during proliferation and in immune cells during acute responses like the germinal centre reaction. This makes Rad18 an attractive tumour treatment target, as it is only expressed and functions in few normal somatic cells.

A multitude of studies have been published showing that Rad18-deficient cell lines show increased sensitivity towards DNA damage^25,26,30–32^. Accordingly, targeting Rad18 in tumours may aim at improved chemotherapy treatment outcomes, rather than direct growth suppression. However, Rad18 has also been found to mediate resistance to oncogene-induced replication stress in tumours^20^ and it may thus affect tumour formation and progression. In line with this finding, formation of hematopoietic tumours upon DMBA treatment was increased, and there was a particular increase in B cell lymphomagenesis ^18^. However, we found no difference in Eµ–Myc mice, a B cell lymphomagenesis model, in the formation of tumours when comparing WT and Rad18KO mice.

One possible explanation for this discrepancy might be that Rad18 is essential for PCNA ubiquitination in yeast and in human HCT116 cells^33,34^, but in some other cell types, Rad18-independent PCNA ubiquitination has been shown^35^. This is apparently due to alternative E3 ligases for PCNA, which have in part been identified^36^. In particular, fibroblasts from Rad18-deficient mice showed residual PCNA ubiquitination^37^, which may limit the effect of Rad18 deficiency in somatic hypermutation, for which PCNA ubiquitination has been shown to be important^14–17^.

Of course, the other Rad18 functions in DNA double strand break repair ^7^ and activation of the Fanconi pathway^8^ may also affect tumorigenesis. Previous studies of Rad18 function in tumorigenesis did not address this issue, so it is not clarified so far. If these Rad18 functions in DNA repair affect tumorigenesis, their role is apparently also redundant in the Eµ–Myc model. However, other models of tumorigenesis, e.g. in solid tumours, may lead to different results, so Rad18 function should be tested in such models.

Taken together, Rad18 has important functions in proliferating cells, where it is highly expressed. However, Rad18 is redundant during B cell lymphomagenesis in the Eµ–Myc model and no difference in overall survival is observed in WT versus Rad18KO mice. Accordingly, the role of PCNA ubiquitination in B cell lymphomagenesis is not clear thus far and should rather be investigated in PCNAK164 mice in which PCNA ubiquitination is definitely defective^15,17^.

## Material and methods

### Mice

Mice with a targeted Rad18 locus and C57BL/6 background were provided by the European Mouse Mutant Archive (B6Dnk;B6Brd;B6N-Tyr^c-Brd^ Rad18^tm1a(EUCOMM)Wtsi^/WtsiCnbc), using the EUCOMM ES clone EPD0027_4_B02. For the deletion of the Rad18 exon2, mice were crossed with delete-Cre mice (B6T(CMV-cre)1Cgn), provided from the University of Cologne^38^, to obtain the Rad18;delCre (RD) mouse line. Additionally, offspring of these crossing were crossed with Flpe mice provided by the Research Centre Lobeda, to obtain the Rad18;DelCre;FlpE (RDF) mice. To generate experimentally used WT and KO mice, RD and RDF mice were bred to be heterozygous for Rad18. To obtain Rad18;delCre; Eµ–Myc (RDM) and Rad18;DelCre;FlpE;Emμ-myc (RDFM) mouse lines, RD and RDF mouse lines were crossed with heterozygous mice regarding Eµ–Myc on a C57BL/6 background^39^, originally provided from Jackson Laboratory. Mice developing tumours were immediately sacrificed via cervical dislocation after reaching humane end point criteria. Mice were bred under pathogen-free conditions and all animal experiments were approved by the Thüringer Landesamt fur Verbraucherschutz. No randomization or blinding was done. All methods were performed in accordance with the relevant guidelines and regulations of the state of Thuringia, Germany.

### Genotyping, mRNA and protein analyses in Rad18 mice

Genotyping was performed with mouse tail cuts of one to two weeks old newborn mice and re-genotyping of experimentally used mice was performed with a mouse tail cut of sacrificed mice. Tissue was lysed with lysis buffer (100 mM Tris HCL pH 8.5, 200 mM NaCl, 5 mM EDTA and 0.2% SDS) and 60 μg/ml protease K. Isolation of DNA was performed with 5 M NaCl and purified with 100% isopropyl and 70% ethanol. Storage of DNA samples employed TE buffer (10 mM Tris pH 8.0 and 1 mM EDTA) at −20 °C.

Testis were isolated and lysed with 1.3 mg/ml collagenase D in 1 × PBS and cell suspensions were generated. For MEFs, E13,5 embryos were isolated of sacrificed mice and dissected. 0.5% trypsin was added and a cell suspension was generated. Cells were seeded and washed two days after seeding to generate fibroblast single cell suspensions. For protein analysis, Western Blot was performed using testis lysate as well as lysates of MEFs and the HCT116 cell line or lysates of primary murine B cells stimulated with anti-CD40/IL-4 as well as DG75 and Raji cell lysates as positive controls. Samples were loaded onto 10% SDS gels. The primary antibodies α-Rad18 (Ab188283; Abcam, Fig. 2D), α-Rad18 (Ab188235; Abcam, Suppl. Figure S4), α-Actin (A2066; Sigma) and α Vinculin (SAB4200080, Sigma) and the secondary antibody α-rabbit IgG (7074; Cell Signaling) or α-mouse IgG (W402B, Promega) were used. TBST was used for washing and blocking, 1 × PBS for actin antibody incubation. For RT-PCR, cDNA synthesis of testis samples was prepared with a cDNA synthesis kit (11,483,188,001; Roche / Merck). For all primers and PCR cycler programs, see Suppl. Figure S1.

### Immunofluorescence on cells and tissue sections

Splenic B cells were isolated from single-cell suspensions of spleens of mice sacrificed by cervical dislocation using a MACS depletion with α-CD43 beads (Miltenyi Biotec.) Sterile coverslips were covered with Poly-L-lysine (Sigma) and dried out. Cells were seeded on coverslips and incubated for 1 h, 37 °C and 5% CO_2_. Cells were fixed with 4% formaldehyde (FA) and permeabilised with ethanol. Cells were stained with α-Ki-67 (RM-9106-S1; Thermo Fisher Scientific), α-Rad18 (ab188283; abcam); α-mouse / α-rabbit-Cy3 (115-165-003 / 111-165-003; Jackson IR) and DAPI (Sigma). All washing steps were performed with either 1xPBS/0.2% TritonX or 1xPBS.

To prepare cryo-sections, mouse spleens were encased in TissueTek (SA62550-01; Science Services) and 30% sucrose or alternatively fixed with 4% FA and dehydrated in an ascending gradient of 10%, 20% and 30% sucrose solution. Samples were frozen and taken to the Fritz-Lipmann Institute (FLI) Jena; 10 μm cryo-sections were prepared with a cryostat Leica CM3050. Spleen tissue was stained with α-CD21 conjugated with FITC (123407; BioLegend / 561769; BD Biosciences), α-PNA (B-1075; Vector), α-Ki-67 (RM-9106-S1; Thermo Fisher Scientific), α-Rad18 (ab188283; abcam), streptavidin conjugated with AF647 (016-600-084, Jackson IR), α-mouse/α-rabbit-Cy3 (115-165-003 / 111-165-003; Jackson IR) and DAPI (Sigma). All washing steps were performed with either 1xPBS/0.2% TritonX or 1xPBS.

### B cell isolation and stimulation

Splenic B cells were isolated from single-cell suspensions of 8–16 weeks old male and female mice using MACS depletion with α-CD43 beads (Miltenyi Biotec.). Per culture, 5 × 10 ^5^ cells/ml were cultured in RPMI 1640 medium (Invitrogen) with 10% FCS (Sigma), 10 mM HEPES (Thermo Fisher Scientific), 50 mM 2-mercaptoethanol (Sigma) and 0.2 U/ml penicillin/streptomycin (Invitrogen) and stimulated either with 1 μg/ml anti-CD40 (eBiosciences) or 10 μg/ml LPS (Sigma) and in both cases with 20 ng/ml IL-4 (eBiosciences) 1 h after seeding. To analyse proliferation, 1 μM CSFE (Invitrogen) was added prior to seeding and stimulation.

### Flow cytometric analyses

For survival and class switch recombination experiments, mouse spleens of RD/RDF WT and KO mice were isolated and single B cell suspensions of the whole tissue were prepared after MACS depletion as described above. Activated B cells were stained with α-B220-BV785 (103246; BioLegend), α-IgG1-FITC (553443; BD) and TO-PRO3 (Invitrogen). CSFE cultivated, activated B cells of RD mice were stained with α-B220-APC (103212; BioLegend) and DAPI (Sigma).

Mouse spleens of RDM/RDFM WT and KO mice were isolated and cell suspensions of the whole tissue were prepared. Cells were stained with α-CD3-PE (553064; BD), α-B220-BV785, α-B220-PE (553090, BD Biosciences), α-CD19-BV421 (115,549; BioLegend), α-IgM-FITC (553408; BD Biosciences), α-CD138-Pe-Cy7 (142514; BioLegend), Sca-1 (or Ly-6A7E; 108139; BioLegend), and TO-PRO3 or DAPI . Flow cytometry was performed on a LSR Fortessa (BD Biosciences) and analysed in FlowJo (FlowJo, LLC).

### Ethics declaration statement

All experiments involving life animals (mice) were approved by the responsible committee of the Freistaat Thüringen. The study was performed according to the ARRIVE guidelines (https://arriveguidelines.org/arrive-guidelines).

### Supplementary Information

Supplementary Information.

## Data Availability

All data generated or analysed in the course of this study are included in the published article and supplementary files.
