# Supplementary material for: Role of Rad18 in B cell activation and lymphomagenesis
Source: Sci Rep. 2024 Mar 25;14:7066. doi: 10.1038/s41598-024-57018-w (PMC10963733; doi:10.1038/s41598-024-57018-w)
Supplement: Supplementary file 1 — Supplementary Information. [file 41598_2024_57018_MOESM1_ESM.pdf]

A

Rad18 Exon 2 genotyping	Rad18_FRT_fwd3	5' AAGTATAGGAACTTCGTCGAGATA 3'
	Rad18del_fwd3	5' AGGCATTGAGTGTGCTGTTG 3'
	Rad18_rechts_rev1	5' AACCATTTGTCTGTCATCCTGC 3'
Rad18 Cassette genotyping	Rad18_F2	5' TGTGTGCTTACCTGGGATGT 3'
	Rad18_EN2_R2	5' CTTTCTGGCCTCGTTTGCAT 3'
	Rad18_loxP_rev	5' TGTATGCTATACGAAGTTATCTCGAC 3'
	Rad18_R	5' CCTGGCAAACAATTTTCCCTC 3'
Myc genotyping	Emμ1	5' GCTGGGGTATGGATACGCAG 3'
	cMyc1	5' GGTGTAAACAGTAATAGCGCAG 3'
	mRag1.1	5' GCTGATGGGAAGTCAAGCGAC 3'
	mRag1.3	5' GGGAACTGCTGAACTTTCTGTG 3'
Rad18 RT-PCR	e1_fwd (b-c)	5' GGGTTGGCGGTGATGAAG 3'
	e2-3_fwd (a)	5' TAGATGACTTGCTGCGCTGT 3'
	e2-3_rev (a-b)	5' CAACAAGTTGGGCACTGAGTT 3'
	e5_rev (a+c)	5' CAGTCACCAGTTTCTCTAATCAGG 3'
	β-actin qRT fwd	5' AGTGTGACGTTGACATCCGT 3'
	β-actin qRT rev	5' TGCTAGGAGCCAGAGCAGTA 3'

B

Exon 2 – Rad18

3 min	94°C	
45 s	94°C	x35
30 s	60°C	
1 min	72°C	
10 min	72°C	
∞	4°C	

Cassette – Rad18

3 min	94°C	
45 s	94°C	x35
30 s	65°C	
1 min	72°C	
10 min	72°C	
∞	4°C	

Myc

5 min	94°C	
30 s	94°C	x35
30 s	61.5°C	
45 s	72°C	
5 min	72°C	
∞	4°C	

Exon 2 – RT-PCR

5 min	94°C	
30 s	94°C	x40
30 s	58°C	
45 min	72°C	
5 min	72°C	
∞	4°C	

Actin – RT-PCR

5 min	94°C	
30 s	94°C	x35
30 s	58°C	
45 s	72°C	
5 min	72°C	
∞	4°C	

Supplementary Figure S1: PCR primers and programs for genotyping and RT-PCR

A) Primers used for genotyping of RD, RDF, RDM and RDFM mice and RT-PRC primers for Rad18 exons and actin as a loading control. **B)** Cycler programs for all sequence analyses for RD, RDF, RDM and RDFM genotyping as well as Rad18 exon RT-PCR and actin as a control.

A

Figure 2B upper gel

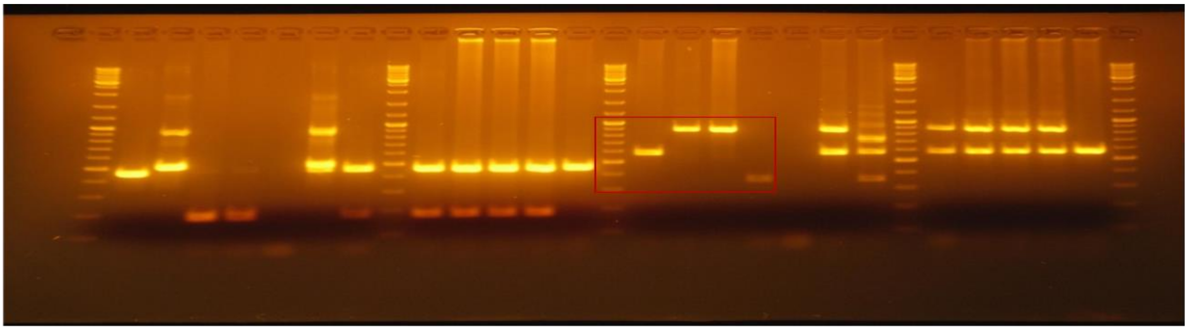

Cassette confirmation

Figure 2B lower gel

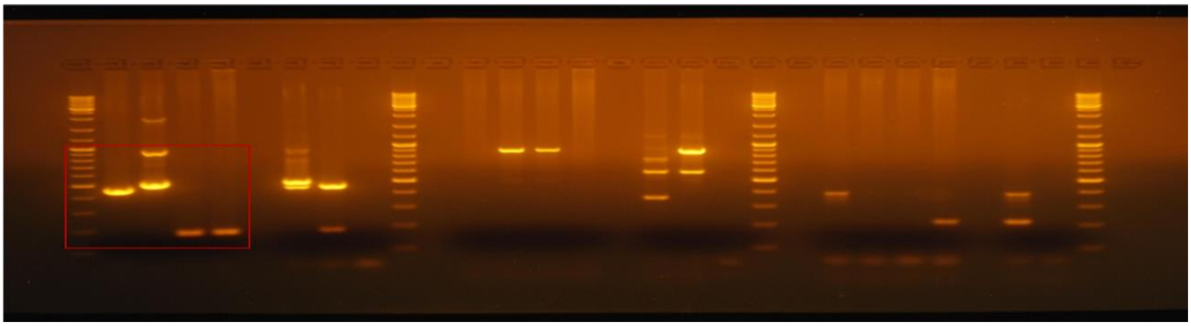

Exon2 confirmation

B

Figure 2C gel b | Figure 2C gel a

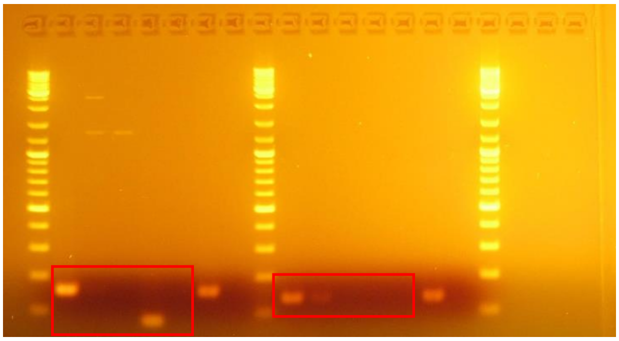

Figure 2C gel c

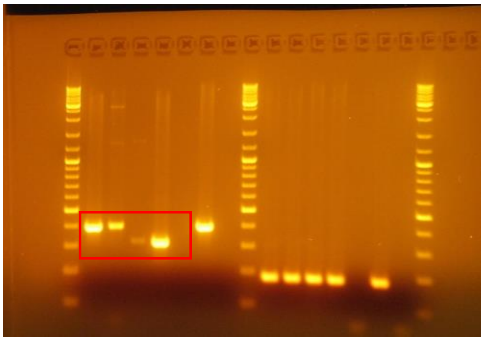

Figure 2C actin gel

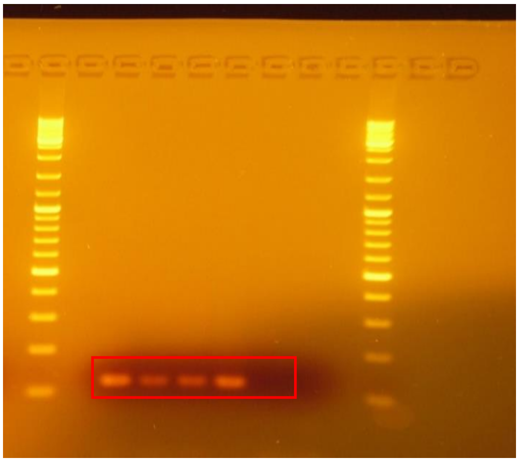

Supplementary Figure S2: Uncropped Western Blots and Ponceau staining

A) Uncropped PCR gels shown in Figure 2B. Red border indicates region of interest used in Figure 2B. **B)** Uncropped RT-PCR gels shown in Figure 2C. Red border indicates region of interest used in Figure 2C.

α-Rad18 o.n. (3min ECL)
[ab188283]

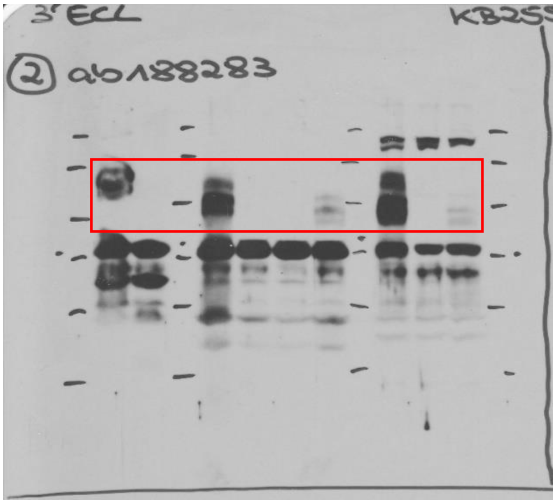

α-Rad18 o.we (5min ECL)
[ab188283]

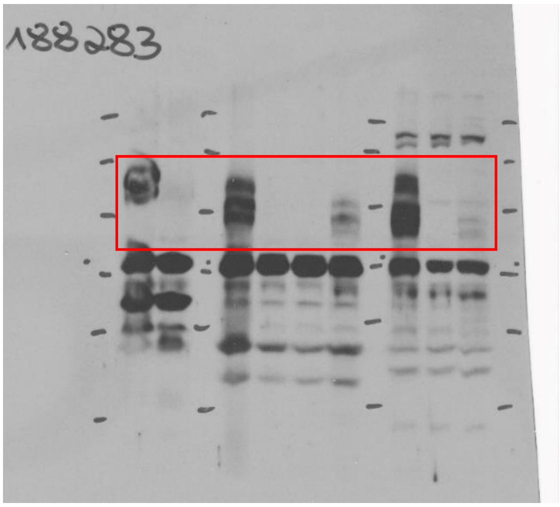

α-Actin (4min ECL)

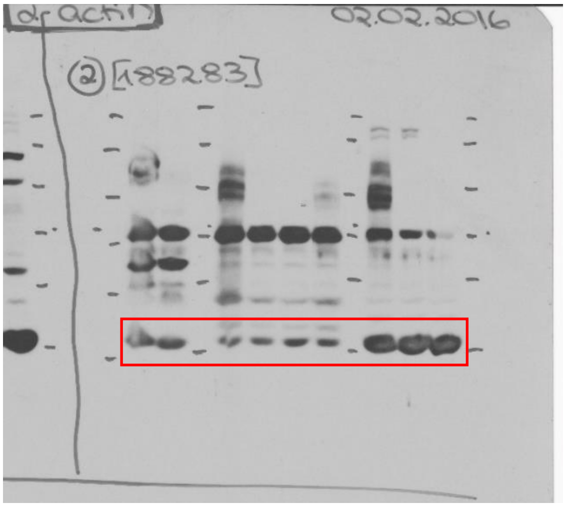

Ponceau

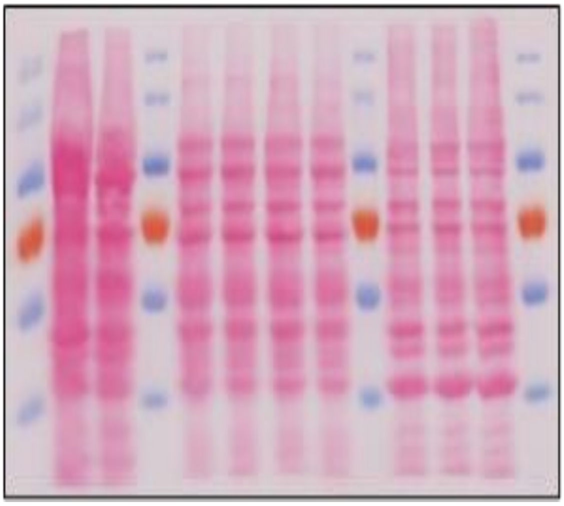

Supplementary Figure S3: Uncropped Western Blots and Ponceau staining

Uncropped Western Blots and the Ponceau staining as a control for consistent protein concentrations and loading correspond to Figure 2D. Samples were incubated with primary AB overnight (o.n.) or over weekend (o.w.). Actin was used as a loading control. Red border indicates region of interest used in Figure 2D.

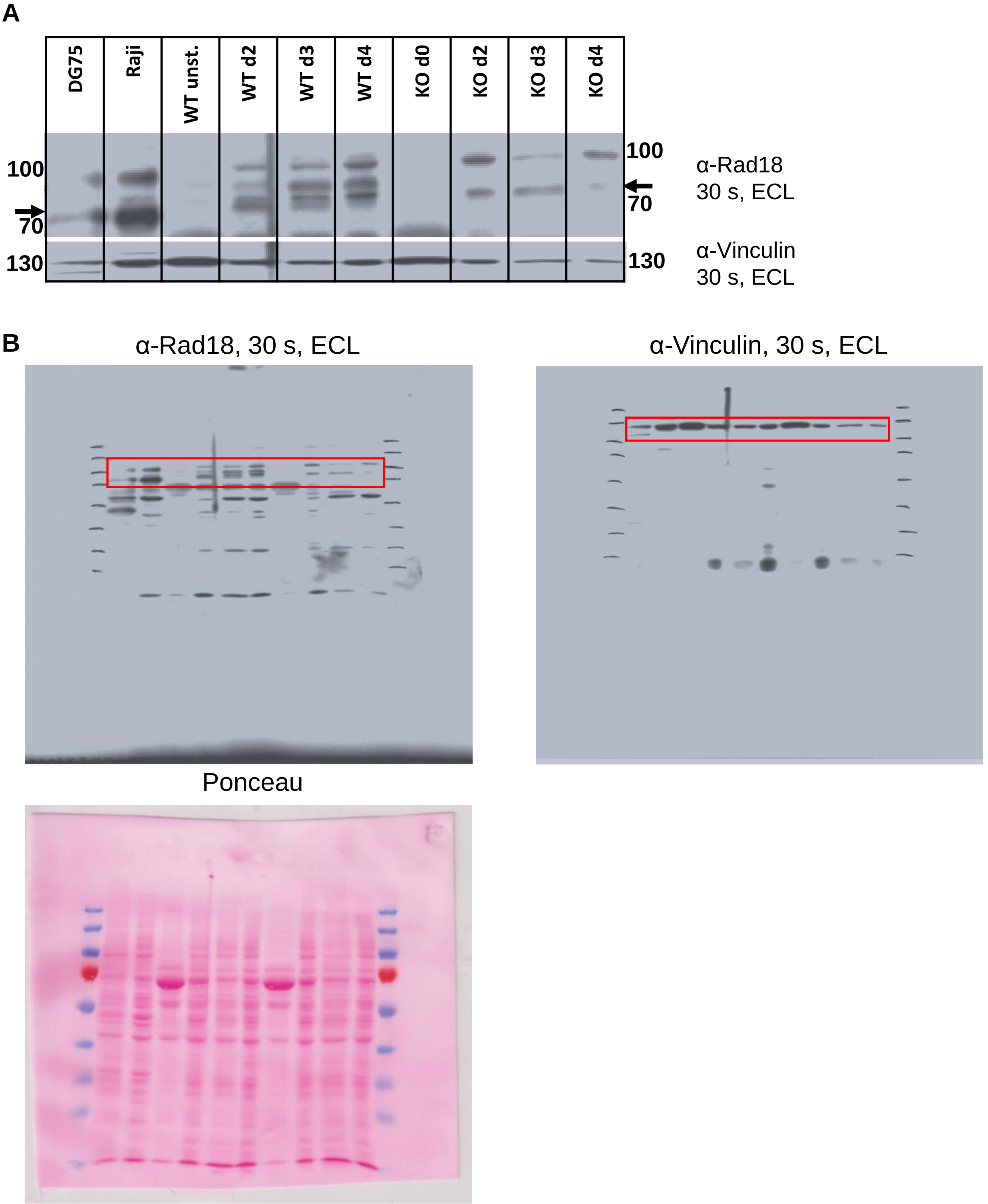

Supplementary Figure S4: Western Blot and Ponceau of murine, activated B cells

A) Western Blot of unstimulated, primary, murine B cells and stimulated B cells of Rad18 WT and KO (RDFM) with α-CD40 and IL-4 for 2, 3 and 4 days as well as DG75 and Raji cells as positive controls. Arrows indicate band of interest. Vinculin was used as loading control. **B)** Uncropped Western Blots and Ponceau staining as a control for consistent protein concentrations and loading correspond to A). Red border indicates region of interest used in A).
